# Supplementary material for: Perceived transcultural self-efficacy and its associated factors among nurses in Ethiopia: A cross-sectional study
Source: PLoS One. 2021 Jul 22;16(7):e0254643. doi: 10.1371/journal.pone.0254643 (PMC8297891; doi:10.1371/journal.pone.0254643)
Supplement: S2 File — (DOCX) [file pone.0254643.s002.docx]

**Interview translation**

| **Participant 1**  **Age: 28**  **Sex: Male** | Patients come from different places, like southern region, Gambela, and Sudan. However, I individually have the confidence of medium type though there are some disagreements among some few things. Above all, I become happy when I see my patients have received all they want. Therefore, I always forget my ethnical background and I think as if I belong to all whenever I get up for caring. I do have a very good confidence even if there are challenges in such culturally different patients. For instance, I look for somebody who translates the language I do not know when I care for patients speaking language strange to me. And also respect their religions every time I am with them. Yea, giving care that has culture at its centre needs determination. You know being a nurse teaches you many things. Communication skill is very important and I have been improving it since I was recruited here. In addition one’s desire and motivation play a great role for the care of patients since they may come from many cultures. I am willing and motivated to serve these people. Millions of people come to this hospital. But it is not by separating them by race, ethnicity, tradition, value, and belief that you care… therefore, since I am motivated to deliver care in diversity, my perception of confidence in such condition is very good. |
| --- | --- |
| **Participant 2**  **Age: 35**  **Sex: male** | Caring for the multicultural population is quite different from caring for those who are uni-cultural. You know populations served at this institution belong to many cultures and this brings a lot of challenges. Despite these challenges I imagine my confidence to be medium. Therefore, I am sure that I am on the right way of handling multicultural patients though I could not say patients get treated having their cultural needs met as exactly as they want.  Many things influence confidence when I care for the kinds of clients I mentioned above. I know that I must be familiar with their languages, religion, life styles and ways of thinking. But these could not be achieved all the time. Therefore, it is obvious that one’s confidence is touched by these things. I do have a strong desire of seeing the clients happy because they get their need. And also there is motivation in me to handle patients belonging to different cultures. I sure that this internal motivation has helped me a lot. If I had lacked such motivation, truly my confidence would have been very low. In the world of caring, taking culture as the sensitive issue is important. Because if a nurse considers all their patients as if they belong to one culture, they leave the patients without getting their needs. So one should look at the back of all the patients first before getting into care. |
| **Participant 3**  **Age: 31**  **Sex: Male** | I see my confidence of caring for patients of different cultural backgrounds to be of medium type. As it is known the hospital covers more than 15 million people within its catchment area. These people come from different ethnicity, religion and the like. Therefore there are different way of thinking, different values and cultures within these people. Therefore there are many things which affects our confidence. For example, one is related with language. If you do not know the language the patient speaks, the option you have to find someone who knows that language and if you do not get, your confidence is affected. Ability to communicate with people, including the staff, and having strong communication skill through the diverse cultures affects nurses’ confidence of delivering care in order meet the needs of the clients. The more I, as a nurse, have good communication skill, the more I am confident. However, the strongest thing that challenges nurses face is developing their communication skill because of diversity of language spoken here in the hospital. You know this hospital gives care for a lot of patients and for example patients come from different regions speaking different languages and having different cultures. This expands your motivation and lets you to the way to handle such diversity. When you find the way, your confidence is increased. |
| **Participant 4**  **Age: 25**  **Sex: Female** | I see caring for such culturally diversified people to have a lot of challenges. However, I perceive confidence for caring to be of medium type. This confidence can be affected by many things. For instance, the language the patient speaks puts challenges to caring. You know there is no any interpreter recruited here in this hospital. Therefore, when you have patients speaking the languages you do not know, you look for somebody to help you. This negatively affects one’s confidence. On the other hand, this also reduces one’s communication ability and results in hesitation of one’s confidence. Another thing which affects my confidence is experience. My confidence in delivering care to patients of different cultures is increased as I stay longer in caring practice. Again, as I am able to communicate effectively amid the diversity of language, thoughts, religion, lifeways and etc, I deliver the care for which you are responsible with confidence. Therefore, considering culture matters a lot.  You must know the culture of the patients and accept their cultures. You do this when you are sensitive to the culture of your patients. This improves your cultural confidence because you consider it sensitive and act accordingly. Above, having motivation is very important. If you have motivation to work, there is nothing which prevents you from achieving your goal. Having motivation to deliver care for culturally diverse group of patients, positively affects nurses’ confidence. |
| **Participant 5**  **Age: 26**  **Sex: Female** | I respect every cultural and religious practices of patients whenever I care for them. I am delighted whenever I see my patients received all they need and improved. But one that makes seeing this hard is the diversity of patients coming here in culture. That strong ambitions and motivation of mine, seeing my patients happy, helped me to find the way to overcome the challenges culture created. To tell you the truth, it is a hard task but I imagine it to be medium because there are different cultures and different ways of thinking. Thus if you do not accept this, later it would influence your confidence.  There are many things which affect nurses’ confidence in such diversified patients, either negatively or positively. One thing may be the language they speak. When I am not familiar with language, I do not deliver care confidently the way I do for those with which I am familiar. Even when I have somebody to tell to the patient what I want to tell, I am not satisfied as when do it myself. Care needs collaboration with other workers and to communicate with them language matters a lot. When I communicate very well, I can solve the problems quickly. Therefore, having good communication ability is crucial to give care with confidence As tried to tell you earlier, having motivation for doing things in such diversity affects confidence. I have motivation means I do my task confidently. |
| **Participant 6**  **Age: 32**  **Sex: Male** | Many patients come to this hospital for getting health care. Some of them from Jimma town and from its surroundings, some from Southern Nations, Nationalities and People’s Region, some from Gambela Region and some few from Sudan. These people have their own cultural beliefs, thoughts and practices. I hope you understand how hard is working in such conditions. But the nature of our work forces us to closely work with patients. Therefore, nurses must be aware of all these cultures and keep in mind their cultures whenever they approach patients specifically. If a nurse does not do that, his/her confidence is greatly and negatively influenced. Therefore, as nurses are more and more culturally sensitive, they tend to have higher confidence in giving care to patients coming from different cultures. To deliver care according to patients’ culture, nurses’ must have first motivation to do that. If you are not motivated, you finish your task seeing them as if they are same. At that time, patients left dissatisfied might be there. When you detect this, your confidence is negatively affected.  When you stay longer in caring practice you develop more and more confidence when compared to that you have at the beginning. This could be due to many things: you get familiar with their languages, religious, beliefs, thoughts and cultural practices. |
| **Participant 7**  **Age: 30**  **Sex: Female** | I perceive my cultural confidence is very good. I come in contact with many patients daily and I have worked as nurse for many years. Things seeming hard at the very beginning, became easily manageable as days passed. The reasons are: cultural understanding gets improved from time to time, your motivation gets better, you see others managing the same condition and getting familiar with the languages. Therefore, I can say that, from my experience, those nurses who worked for many years might have greater confidence when they care for patients belonging to many cultures. One factor influencing confidence could be this.  Considering cultural background in caring profession is very important. Many patients, millions, come to get care. Therefore, nurses must be knowing that they should be familiar with and sensitive to cultures and give care according to the patients’ culture. When they do this, their cultural confidence gets better. So this could be another factor which can influence cultural confidence.  The nature of the nurses work makes them t0o be very cooperative. Nurses care for patients, many times, in collaboration with other health care providers. As for me, I do have very good communication skill that made me very cooperative. Therefore, I can simply handle the problems there to do my task and I am very confident in my work because of this. So I have no hesitation if say strong communication ability is linked with strong confidence. And also ability to communicate among patients having different cultures influences confidence for caring. |
| **Participant 8**  **Age: 26**  **Sex: Female** | I imagine my confidence to be medium even if there are many challenges present because of diversity of culture. More than 15 million people are served at this hospital coming from many places. My care cannot satisfy all these people if my care is not specific to their cultures. Unfortunately, it is very hard to adjust perfectly to all the cultures present because it is hindered by many things. First, you may not know every languages the patients speak, you may not know all the practices religiously prohibited and you need time to be familiar with all these. Second, presence of many patients makes nurses busy and unable to effectively communicate with patients. When this happen nurses get into work without understanding cultural needs of all their patients. Lastly, nurses desire to work among such diversity puts influence to adjusting care according to culture. To sum up, nurses must be very strong and confident because the work they do is very hard. |
| **Participant 9**  **Age: 32**  **Sex: male** | I imagine my cultural confidence is very good. This hospital gives service to about 15 million people. They may come from outside Oromia region and even from outside Ethiopia. Each come with different cultural background and this makes caring hard. Therefore, nurses must be devoted and motivated, must spend their time with patients to get familiar with beliefs, lifeways, languages and ideas of their patients. To speak frankly, it is difficult to deliver care that perfectly matches with cultural needs of the patients. However, one can improve his/her confidence through time. Experience teaches the nurse many things and strongly affects cultural confidence. This is because you get familiar with many things as you stay more and more years in this profession. The factors which influence nurses’ confidence in such conditions could not be limited to this. Many other factors might be there. For example, I can raise factors like language barrier, communication ability and motivation. When I find patients speaking the language I do not know, my confidence gets lower. The reason behind this is that I must communicate each and every thing I do with patient. I could not that when there is language barrier. The other is communication ability. I am very cooperative and able to communicate effectively if no barrier. This helps me to have strong confidence. But, you know, some people suffers a lot because they lack this. In the world of work, motivation does great things. It role in nursing profession is greatest, I can say. Therefore, having desires and motivation to work for patients diversified by culture positively affects confidence of nurses. |
| **Participant 10**  **Age: 27**  **Sex: Female** | I perceive my confidence to be good when I care for patients with different cultural backgrounds. You know patients come to Jimma from many areas. Different kinds of people come and we are adjusting our care in accordance with their difference even if it is hard. We do this because, above all, we want our patients get satisfied. Therefore, I can say my cultural confidence is good. But it is affected by many things. As I tried to tell above, number of patients are many (patient flow is high. When the number of patients become many, giving culturally appropriate care is hard. If you do not give that, your confidence might get lower. The other factor could be language. If you are not familiar with language of your patient, you may not of strong confidence. But we are trying to improve this by having somebody around translate the language. The other factor might be considering presence of many cultures as the sensitive issue. If you do that, you search for all the ways there to achieve your goal. If not, if see it as the hardest thing and you will not have strong confidence because you fear. Again, care needs very cooperative and communicate nurses. Nurses communicate with patients, attendants and staffs when they give care, therefore they must a strong communication ability. Nurses who wave low communication ability may not be as confident as those who have strong communication ability. |
