# Supplementary material for: Perceived transcultural self-efficacy and its associated factors among nurses in Ethiopia: A cross-sectional study
Source: PLoS One. 2021 Jul 22;16(7):e0254643. doi: 10.1371/journal.pone.0254643 (PMC8297891; doi:10.1371/journal.pone.0254643)
Supplement: S3 File — (DOCX) [file pone.0254643.s003.docx]

1. **Quantitative Data Collection Tools**

**Participant Information Sheet**

**Title of the study:** Perceived Transcultural Self-efficacy and its Predictors among Nurses Working at Jimma Medical Center, Oromia Region, Southwest Ethiopia

**Name of investigators and institution:** Mr. Robera Demissie (Jimma University), Mr. Abebe Abera (Jimma University), Mesfin Beharu (Jimma University), Shuma Gosha (University of Gondar)

**Name of sponsor:** Jimma University, Ethiopia

**Approval:** The study has been approved by Jimma University Ethical institutional Review Board.

**Introduction**

You are invited to participate in a research study. Before you decide, you need to understand why the research is being done and what it involves. Please take your time to read and consider this information carefully before you decide. Ask questions if anything you read is unclear or if you would like more information. After you are properly satisfied that you understand this study, and that you wish to participate, you must sign the informed consent form on the next page.

**Purpose of the study:** The purpose of this study is to assess perceived transcultural self-efficacy and its predictors among nurses working at Jimma Medical Center. The findings from the study will improve awareness of nurses related to transcultural self-efficacy and provide evidence-based nursing care to culturally and ethnically diverse clients, this in turn improves the quality of care.

**Participation:** Your participation in this study is voluntary. If you do not want to, you should not be in this study. You may also refuse to answer any questions you do not want answered.

**Right to withdraw:** If you volunteer to be in this study, you may withdraw from it at any time. Your refusal to participate or withdrawal will not affect you in any way.

**Benefit and risk:** The study may not benefit you directly. There is no harm or discomfort including physical, psychological, and social risks to you except the time spent for filling the questionnaire.

**Study procedures**: You are approached to the study team member(s) who will explain the
details of the study to you and ask for your consent to participate in the study. If you are
willing to participate in this study, you will be asked to sign on a consent form.

**Contact person:** If you have any question related to the study or if you would like to be informed of the results after its completion, you can call the investigators on +251931213709 (Mr. Robera), +251913053500 (Mr. Abebe), +251913872267 (Mr. Mesfin) or +25135054730 (Mr. Shuma) without any hesitation or you can email them at [roobeeraadb@gmail.com](mailto:roobeeraadb@gmail.com), [abeef2011@gmail.com](mailto:abeef2011@gmail.com), [laliistuubm@gmail.com](mailto:laliistuubm@gmail.com) or [shumagosha33@gmail.com](mailto:shumagosha33@gmail.com).

**Thank you very much for your cooperation!**

**Consent Form**

I freely, voluntarily, and without the element of force or coercion consent to be a participant in the research study entitled “***Perceived Transcultural Self-Efficacy and Its Predictors among Nurses Working at Jimma Medical Center, Oromia Region, Southwest Ethiopia***.”

By signing this form, I confirm that:

- I have been given oral and written information for the above study and have read and understood the information given.
- I have had sufficient time to consider participation in the study and have had the opportunity to ask questions and all my questions have been answered satisfactorily.
- I understand that my participation is voluntary and I can freely withdraw from the study at any time without giving a reason and this will in no way affect me.
- I understand the risks and benefits, and I freely give my informed consent to participate in the conditions stated.
- I understand that my answers to the questions will not be given to anyone else and no reports of the study ever identify me in any way. I understand that all personal details will be treated as strictly confidential.
- I understand that participation in the study does not involve risks.
- I understand that I may contact Robera Demissie at +251931213709 or Abebe Abera at +251913053500 Mesfi Beharu at +251913872267 or Shuma Gosha at +25135054730 and I may email them at [roobeeraadb@gmail.com](mailto:roobeeraadb@gmail.com), [abeef2011@gmail.com](mailto:abeef2011@gmail.com), [laliistuubm@gmail.com](mailto:laliistuubm@gmail.com) or [shumagosha33@gmail.com](mailto:shumagosha33@gmail.com) for answers to any questions that may arise about this research study or my rights. Furthermore, I am aware that I can request and receive a copy of the study results.

___________________ ______________

**Participant’s Signature Date**

___________________ ______________ ______________

**Data collector’s name Signature Date**

**Definition of terms that might help you fill questionnaires**

**Ethnocentrism:** Act of judging another culture and believing that the values and standards of one's own culture are superior especially with regard to language, behavior, customs, and religion

**Genogram:** A record that contains family history (name of each of the family members and their relationship to the family, including diseases and conditions that occur within the family).

**Other**: Rate the points under ‘other’ by considering averagely the culture of ethnic groups you know but not listed in the items or group categories.

**Structured Questionnaire**

**Part I: Socio-demographic Information**

| **No** | **Questions** | **Answers** | **Remark** |
| --- | --- | --- | --- |
| 101 | Age | ________ |  |
| 102 | Sex | 1. Male 2. Female |  |
| 103 | Ethnicity | 1. Oromo 2. Amhara 3. Dawro 4. Other (specify)_________ |  |
| 104 | What is your religion | 1. Orthodox 2. Muslim 3. Protestant 4. Others (specify)___________ |  |
| 105 | What is your highest level of education: | 1. Diploma nurse 2. Bachelor’s degree nurse 3. Master’s degree nurse |  |
| 106 | Your monthly income | __________ETB |  |
| 107 | Years of nursing experience | __________months/years |  |
| 108 | What is your current role (professional role). In the last 3 months | 1. Matron nurse 2. Supervisor nurse 3. Head nurse 4. Inpatient nurse 5. Outpatient nurse |  |

**Part II: Cultural Self-efficacy scale**

| **CONFIDENCE IN MY KNOWLEDGE OF CULTURAL CONCEPTS** | | | | | | | | | | | | |
| --- | --- | --- | --- | --- | --- | --- | --- | --- | --- | --- | --- | --- |
| **No** |  | **Very little confidence** | **Little confidence** | | | **Neutral confidence** | | **Moderate confidence** | | | **High confidence** | |
| **201** | Distinguishing between inter and intracultural diversity | 1 | 2 | | | 3 | | 4 | | | 5 | |
| **202** | Distinguishing between ethnocentrism and Discrimination | 1 | 2 | | | 3 | | 4 | | | 5 | |
| **203** | Distinguishing between ethnicity and culture | 1 | 2 | | | 3 | | 4 | | | 5 | |
| **CONFIDENCE IN MY KNOWLEDGE OF CULTURAL PATTERNS**  Indicate your confidence on your knowledge concerning each of the following ethnic groups: | | | | | | | | | | | | |
| **Groups** | | **Oromo** | | **Amhara** | | | **Dawro** | | | **Other** | | |
| **204** | Family Organization | 1 2 3 4 5 | | 1 2 3 4 5 | | | 1 2 3 4 5 | | | 1 2 3 4 5 | | |
| **205** | Role Differentiation | 1 2 3 4 5 | | 1 2 3 4 5 | | | 1 2 3 4 5 | | | 1 2 3 4 5 | | |
| **206** | Child Care Practices | 1 2 3 4 5 | | 1 2 3 4 5 | | | 1 2 3 4 5 | | | 1 2 3 4 5 | | |
| **207** | Utilization of Health System | 1 2 3 4 5 | | 1 2 3 4 5 | | | 1 2 3 4 5 | | | 1 2 3 4 5 | | |
| **208** | Types of Social Supports | 1 2 3 4 5 | | 1 2 3 4 5 | | | 1 2 3 4 5 | | | 1 2 3 4 5 | | |
| **209** | Utilization of Traditional Folk Health Practices | 1 2 3 4 5 | | 1 2 3 4 5 | | | 1 2 3 4 5 | | | 1 2 3 4 5 | | |
| **210** | Nutritional Patterns | 1 2 3 4 5 | | 1 2 3 4 5 | | | 1 2 3 4 5 | | | 1 2 3 4 5 | | |
| **211** | Economic Style of Living | 1 2 3 4 5 | | 1 2 3 4 5 | | | 1 2 3 4 5 | | | 1 2 3 4 5 | | |
| **212** | Migration Patterns | 1 2 3 4 5 | | 1 2 3 4 5 | | | 1 2 3 4 5 | | | 1 2 3 4 5 | | |
| **213** | Social Class Structure | 1 2 3 4 5 | | 1 2 3 4 5 | | | 1 2 3 4 5 | | | 1 2 3 4 5 | | |
| **214** | Employment Patterns | 1 2 3 4 5 | | 1 2 3 4 5 | | | 1 2 3 4 5 | | | 1 2 3 4 5 | | |
| **215** | Patterns of Disease/Illness | 1 2 3 4 5 | | 1 2 3 4 5 | | | 1 2 3 4 5 | | | 1 2 3 4 5 | | |
| **216** | Beliefs About Health and Illness | 1 2 3 4 5 | | 1 2 3 4 5 | | | 1 2 3 4 5 | | | 1 2 3 4 5 | | |
| **217** | Beliefs Towards Respect and Authority | 1 2 3 4 5 | | 1 2 3 4 5 | | | 1 2 3 4 5 | | | 1 2 3 4 5 | | |
| **218** | Beliefs Toward Authority | 1 2 3 4 5 | | 1 2 3 4 5 | | | 1 2 3 4 5 | | | 1 2 3 4 5 | | |
| **219** | Religious Beliefs and Patterns | 1 2 3 4 5 | | 1 2 3 4 5 | | | 1 2 3 4 5 | | | 1 2 3 4 5 | | |
| **CONFIDENCE IN THE FOLLOWING SPECIFIC NURSING SKILLS** | | | | | | | | | | | | |
| **220** | Using an interpreter ( using any person around for translation) | 1 | 2 | | 3 | | | | 4 | | | 5 |
| **221** | Entering into an ethnically distinct community | 1 | 2 | | 3 | | | | 4 | | | 5 |
| **222** | Defense of patient’s rights (advocacy) | 1 | 2 | | 3 | | | | 4 | | | 5 |
| **223** | Performing a 24 hour diet review | 1 | 2 | | 3 | | | | 4 | | | 5 |
| **224** | Taking a life history | 1 | 2 | | 3 | | | | 4 | | | 5 |
| **225** | Developing a genogram | 1 | 2 | | 3 | | | | 4 | | | 5 |

**Part III: Intercultural communication scale (ICS)**

| **S/N** | **Items** | **Strongly disagree** | **Disagree** | **Neutral** | **Agree** | **Strongly agree** |
| --- | --- | --- | --- | --- | --- | --- |
| **301** | I understand the feelings of patients from other cultures | 1 | 2 | 3 | 4 | 5 |
| **302** | I communicate well with patients from other cultures | 1 | 2 | 3 | 4 | 5 |
| **303** | I can easily resolve misunderstandings with patients from other cultures | 1 | 2 | 3 | 4 | 5 |
| **304** | I understand the point of view of patients from other cultures | 1 | 2 | 3 | 4 | 5 |
| **305** | I can empathize with patients from other cultures | 1 | 2 | 3 | 4 | 5 |

**Part V: Cultural sensitivity scale (CSS)**

| **S/N** | **Items** | **Strongly disagree** | **Disagree** | **Neutral** | **Agree** | **Strongly agree** |
| --- | --- | --- | --- | --- | --- | --- |
| **401** | I know a lot about my patients’ culture | 1 | 2 | 3 | 4 | 5 |
| **402** | I adapt my treatment according to patients’ culture | 1 | 2 | 3 | 4 | 5 |
| **403** | I consider patients’ culture when making recommendations | 1 | 2 | 3 | 4 | 5 |

**Part IV: Interpersonal communication scale (IPCS)**

| **S/N** | **Items** | **Strongly disagree** | **Disagree** | **Neutral** | **Agree** | **Strongly agree** |
| --- | --- | --- | --- | --- | --- | --- |
| **501** | I encourage others to tell me how they feel | 1 | 2 | 3 | 4 | 5 |
| **502** | People tell me that I am easy to talk to | 1 | 2 | 3 | 4 | 5 |
| **503** | Strangers often approach and start talking to me | 1 | 2 | 3 | 4 | 5 |
| **504** | People tell me I am a good listener | 1 | 2 | 3 | 4 | 5 |
| **505** | I am honest with others about my thoughts and feelings | 1 | 2 | 3 | 4 | 5 |
| **506** | I believe that communication will be productive | 1 | 2 | 3 | 4 | 5 |
| **507** | I use examples to help me explain what I am talking about | 1 | 2 | 3 | 4 | 5 |

**Part VI: Cultural motivation scale**

| **S/N** | **Items** | **Strongly disagree** | **Disagree** | **Neutral** | **Agree** | **Strongly agree** |
| --- | --- | --- | --- | --- | --- | --- |
| **601** | I enjoy interacting with people from different cultures | 1 | 2 | 3 | 4 | 5 |
| **602** | I am confident that I can socialize with locals in a culture that is unfamiliar to me | 1 | 2 | 3 | 4 | 5 |
| **603** | I am sure I can deal with the stresses of adjusting to a culture that is new to me | 1 | 2 | 3 | 4 | 5 |
| **604** | I enjoy living in cultures that are unfamiliar to me | 1 | 2 | 3 | 4 | 5 |
| **605** | I am confident that I can get accustomed to the shopping conditions in a different culture | 1 | 2 | 3 | 4 | 5 |

1. **Qualitative Data Collection Tools**

**Consent Form for In-depth Interview**

**Research project title:** Perceived Transcultural Self-Efficacy and Its Predictors among Nurses Working at Jimma Medical Center, Oromia Region, Southwest Ethiopia

**Research investigators:** Robera Demissie, Abebe Abera, Mesfin Beharu and Shuma Gosha

**Approval:** This research has been reviewed and approved by the Jimma University Institutional Ethical Review Board

The interview will take 20 – 25 minutes. I do not anticipate that there are any risks associated with your participation, but you have the right to stop the interview or withdraw from the research at any time.

Thank you for agreeing to be interviewed as part of the above research study. Ethical procedures for academic research require that interviewees explicitly agree to be interviewed and how the information contained in their interview will be used. This consent form is necessary for us to ensure that you understand the purpose of your involvement and that you agree to the conditions of your participation. Would you, therefore, read the accompanying information sheet and then sign this form to certify that you approve the following:

- The interview will be recorded and a transcript will be produced
- You will be sent the transcript and allowed to correct any factual errors
- The transcript of the interview will be analyzed by the research investigator
- Access to the interview transcript will be limited to the researcher
- Any summary interview content, or direct quotations from the interview, that are made available through academic publication or other academic outlets will be anonymized so that you cannot be identified, and care will be taken to ensure that other information in the interview that could identify yourself is not revealed
- The actual recording will be (kept or destroyed state what will happen)
- I agree to be quoted directly if my name is not published and a made-up name is used
- I agree that the researchers may publish documents that contain quotations by me
- Any variation of the conditions above will only occur with your further explicit
  approval

By signing this form, I agree that;

- I am voluntarily taking part in this project. I understand that I don’t have to take part, and I can stop the interview at any time;
- The transcribed interview or extracts from it may be used as described above;
- I have read the Information sheet;
- I don’t expect to receive any benefit or payment for my participation;
- I can request a copy of the transcript of my interview and may make edits I feel necessary to ensure the effectiveness of any agreement made about confidentiality;
- I have been able to ask any questions I might have, and I understand that I am free to contact the researcher with any questions I may have in the future.

____________________ _______________

**Participant’s Signature Date**

______________________ ________________

**Data collector’s Signature Date**

***Contact information***

If you have any further questions or concerns about this study, please contact:

Name of researchers: Robera Demissie, Abebe Abera, Mesfin Beharu and Shuma Gosha

Full address: Jimma, Oromia, Ethiopia

Tel: +251931213709, +251913053500, +251913872267, +25135054730

E-mail: [roobeeraadb@gmail.com](mailto:roobeeraadb@gmail.com), [abeef2011@gmail.com](mailto:abeef2011@gmail.com), [laliistuubm@gmail.com](mailto:laliistuubm@gmail.com) or [shumagosha33@gmail.com](mailto:shumagosha33@gmail.com).

**In-depth Interview Guiding Questions**

**Part I: Socio-demographic characteristics related questions**

1. Sex: _______
2. How old are you?
3. What is your highest level of education in nursing?
4. How many years have you worked as a nurse?
5. What is your current role?

**Part II: Questions regarding transcultural self-efficacy**

1. How do you perceive your confidence while you work with culturally diversified patients?
2. What are the factors that influence your confidence in working with culturally diversified patients?
3. What do you recommend to increase the self-efficacy of nurses working with culturally diversified patients?
