# Supplementary material for: Perceived transcultural self-efficacy and its associated factors among nurses in Ethiopia: A cross-sectional study
Source: PLoS One. 2021 Jul 22;16(7):e0254643. doi: 10.1371/journal.pone.0254643 (PMC8297891; doi:10.1371/journal.pone.0254643)
Supplement: Attachment — Submitted filename: Response to Reviewers.docx [file pone.0254643.s005.docx]

**Response to academic editor and reviewers**

**Which checklist did you use for your study?**

- COREQ checklist was used and it was attached as an additional information

**Please provide detailed description of the sampling strategy, why you choose the method that you have described and why not any other method of sampling, including rationale for the recruitment method.**

- Simple random sampling and purposive sampling were used to select nurses for QAUN and QUAL parts of the study respectively.
- We used simple random sampling because we had sampling frame (list of all the nurses at Jimma medical center. When there is sampling frame, it is better to choose participants using simple random sampling because it produces the representative samples. Systematic random sampling could be applied using this sampling frame but it does not produce samples which are representative as with the method we chose.
- We selected nurses for QUAL part of the study using purposive sampling method because our intention was to have nurses who could give us detailed information and we had predetermined criteria mentioned in the manuscript.

**Please provide a discussion of potential sources of bias**

Researchers work with samples rather than with populations because it is cost-effective to do so. Data from samples can be erroneous and results in sampling bias. Sampling bias refers to the systematic over- or underrepresentation of a population segment. The following is discussion of sources of bias one by one.

- **Social desirability bias**: This occurs when the study respondents want to place themselves at socially acceptable place. Therefore, the most common source of this bias is respondents’ lack of comfort to reveal their attitudes.
- **Recall bias**: a systematic error caused by differences in the accuracy or completeness of the recollections retrieved ("recalled") by study participants regarding events or experiences from the past. This occurs when events over the long period of time is asked.
- **Self-selection bias or volunteer bias**: in studies this offers further threats to the validity of a study as these participants may have intrinsically different characteristics from the target population of the study. Studies have shown that volunteers tend to come from a higher social standing than from a lower socio-economic background.

**Please provide a discussion of limitations**

- Limitations are provided in the final paragraph of discussion part (Page 18)

**Definition of independent and dependent variables are not clear.**

- Sorry for its absence in the first manuscript. Dependent and independent variables are operationally defined in the revised manuscript

**Does the sample size represent the population of Ethiopia proportionality?**

- It does not represent the population of Ethiopia but we used nurses working at Jimma medical center as an accessible population.
- Ten (10) nurses were interviewed to provide data for QUAL part of the study. As it is obvious, small samples are used for qualitative research. Sample size is based on information need in QUAL research

**Why has p<0.25 used in the bivariate analysis**

- In bivariate analysis, independent variables are entered one by one with dependent variable into the bivariate regression model. Then all variables showing significant association in the bivariate analysis are taken together into the multivariable regression model. Variables having insignificant associations at bivariate analysis may show significant association when they are taken together into the multivariable regression model. Therefore, the level of significance should be broadened for bivariate analysis not to miss variables at multivariable analysis. Therefore, p<0.05 leads to missing of variables if used for bivariate analysis. So we used p <0.25 in the bivariate analysis because must literatures recommend this.
