# Supplementary material for: Perceived transcultural self-efficacy and its associated factors among nurses in Ethiopia: A cross-sectional study
Source: PLoS One. 2021 Jul 22;16(7):e0254643. doi: 10.1371/journal.pone.0254643 (PMC8297891; doi:10.1371/journal.pone.0254643)
Supplement: Attachment — Submitted filename: Response to Reviewers.docx [file pone.0254643.s006.docx]

**Response to Academic editor and Reviewers?**

**Response to academic editor**

- I have added some references (Number 16 & 23), Number 16 has been added because I got important information. Number 23 has been added to cite operational definition which was wrongly left.
- Another changes to the reference list is that it has been made complete and correct as per Vancouver referencing style. Some of the references lacked journals, some lacked page numbers, and some lacked volume and issue number. For some, authors’ last name was wrongly written. I reviewed these things and have corrected them. In addition, I have added DOI for many references.

**Response to reviewers**

**It would have been better if a sentence in the abstract was added describing what the score meant**

- I hope this was raised for the statement which talks about transcultural self-efficacy score in the abstract (*The mean transcultural self-efficacy score was 2.89* $\pm$*0.59*). Even if the result part of the abstract lacked this, it has been described in the conclusion part of the abstract that the score means moderate level of self-efficacy.

**Title: requires modification. Hence the study was conducted at one Hospital (JMC), is it possible to say “nurses in Ethiopia”?**

- The title was originally “among nurses working at Jimma medical center.” But there was suggestion to say “nurses in Ethiopia” upon previous review. People served at this medical center is very heterogeneous in culture and ethnicity as is the population of Ethiopia. Ethiopia is multi-ethnic country and there is a lot of diversity in culture. People treated at this medical center is also multi-ethnic and diversified in culture. Therefore, we discussed and considered that suggestion because nurses working here at this medical center can represent nurses working in other parts of the country.

**The word “predictor” is not appropriate for a cross-sectional study. it indicates a great causal association specially used in case of cohort study**

- I changed this word to the word “associated factors” in the revised manuscript

**There is no any statement in the method part of your manuscript which describe the way of sample size determination for appropriate representation of the general population.**

- Sorry for the absence. Now I have added sample size determination in the revised manuscript

**Operational Definitions: what is your reference for your cut of point to say low, middle, or high for TSE?**

- I have added reference in the revised manuscript
